# Active Commuting and Physical Fitness: A Systematic Review

**DOI:** 10.3390/ijerph17082721

**Published:** 2020-04-15

**Authors:** Duarte Henriques-Neto, Miguel Peralta, Susana Garradas, Andreia Pelegrini, André Araújo Pinto, Pedro António Sánchez-Miguel, Adilson Marques

**Affiliations:** 1 CIPER, Faculdade de Motricidade Humana, Universidade de Lisboa, 1649-004 Lisbon and Portugal; duarteneto13@gmail.com (D.H.-N.); mperalta@fmh.ulisboa.pt (M.P.); 2 ISAMB, Faculty of Medicine, University of Lisbon, 1649-004 Lisbon, Portugal; 3 Faculdade de Motricidade Humana, Universidade de Lisboa, 1649-004 Lisbon, Portugal; susanamartinsgarradas@gmail.com; 4 Health and Sport Sciences Center, State University of Santa Catarina, 3664-8600 Coqueiros - Florianopolis, Brazil; pelegrini.andreia@gmail.com (A.P.); andrefsaude@hotmail.com (A.A.P.); 5 Department of Didactics of Music, Plastic and Body Expression, Teacher Training College, University of Extremadura, 10003 Cáceres, Spain; pesanchezm@unex.es

**Keywords:** active commuters, active travel, walking, cycling, physical fitness

## Abstract

Physical fitness (PF) is considered an excellent biomarker of health. One possible strategy to improve PF levels is active commuting. This review, performed accordingly to the Preferred Reporting Items for Systematic Reviews guidelines includes scientific articles published in peer-reviewed journals up to December 2019 that aim at examining the relationship between active travel/commuting and PF. The search was performed in three databases (PubMed, Scopus, and Web of Science). Sixteen studies were included in this review. Findings from the 16 studies were unclear. From the eleven studies on children and adolescents screened, eight were cross-sectional, one prospective cohort, one quasi-experimental, and one experimental. From the five studies on adults, four were experimental and one cross-sectional. Body mass, waist circumference, skinfolds, fat mass, cardiorespiratory fitness, upper and lower strength tests were performed in children, adolescents, and adults. Agility and speed tests were performed only in the young age groups. Majority of the investigations on young ages and adults have shown positive effects or relationships between active commuting and several attributes of PF. However, to avoid misconceptions, there is a need for future robust investigation to identify potential mediators or confounders in this relationship. More robust investigations are essential to understand how and whether decision-makers and public health authorities can use active travel/commuting as a strategy to improve PF in all ages.

## 1. Introduction

Physical inactivity is one of the main risk factors for mortality worldwide [1,2]. Therefore, there is a global need to promote strategies to increase physical activity (PA) levels. PA can be performed in several contexts such as work, organized sports, recreational activities, home activities, and active travel/commuting [2,3,4,5]. Active travel/commuting is an ecological and non-motorized transport mode for all ages, which can be characterized by a form of displacement through PA from/to home and workplace/school. Active commuting increases individual energy expenditure and is easy to incorporate in normal daily routines [6,7]. Active travel/commuting, such as cycling or walking, seems to be an effective strategy to improve daily PA levels; however, it might also improve physical fitness (PF) levels, in addition to promoting health [8,9,10]. Previous studies have demonstrated a strong association between active travel/commuting and PA levels; moreover, higher cardiorespiratory fitness (CRF), strength levels, and lower obesity indicators values have been associated with cycling and walking to school/work in young and adult populations [11,12,13].

PF is considered a biomarker of health, and the most common health-related attributes of PF are CRF, muscular fitness (MF), and body composition [14,15]. Assessing body composition, CRF, and/or MF attributes allows one to monitor an individual’s PA levels and health status, through the performance of most human systems [14]. Previous reviews have examined the relationships between active commuting and several attributes of PF at young ages [16,17,18,19]. Although some positive associations were observed between active commuting and CRF, MF, and body composition, the results are not consistent [20,21,22]. Furthermore, even though there are some studies among adults, there are no systematic reviews examining associations between PF and active travel/commuting in adults [23,24]. For that reason, the relationship between active travel/commuting and PF among several age groups is, thus far, unclear. The aim of this study was to systematically review the evidence on the association between PF and active travel/commuting in both young and adult populations.

## 2. Materials and Methods

This systematic review was performed in accordance to the Preferred Reporting Items for Systematic Reviews and Meta-Analysis (PRISMA) guidelines [25].

### 2.1. Inclusion Criteria

This review includes scientific articles published in peer-reviewed journals until December 31 2019 that aim at examining the relationship between active travel/commuting and PF. Inclusion criteria for articles to be eligible for this review were the following: (1) having a cross-sectional, prospective, observational, cohort, or experimental study design (study design criteria); (2) presenting outcomes of PF, including body composition, CRF, and MF (outcome criteria); (3) examining the association between PF and active travel/commuting (data analysis criteria); (4) focusing on young or adult population (participants criterion); (5) being published in English, Portuguese, or Spanish (language criteria); (6) not having been included in a previous systematic review on the same topic. Articles not meeting all of the inclusion criteria were excluded from the systematic review (exclusion criteria).

### 2.2. Search Strategy

Three international databases were screened, including PubMed, Scopus, and Web of Science, with scientific articles published in peer-reviewed journals until 31 December 2019 aiming at examining the association between active travel/commuting and PF being identified. In each database, a search was performed through a pre-defined combination of keywords sought in the title and abstract of articles. The combination of keywords used was the following: travel* OR transport* OR commut* OR cycle OR cycling OR bicycl* OR bik* OR walk* OR AND fitness* OR physical function OR physiological function OR physical health OR physiological health*. After the search, identified articles were screened for duplicates and were removed if there were duplicates. Then, title and abstract of identified articles were screened by two authors (D.H.-N.; M.P.) in order to identify studies that met all the inclusion criteria. After screening, articles identified as relevant were retrieved for full text analysis. Full text articles were examined by three authors (D.H.-N.; M.P.; A.M.) for inclusion in the systematic review, and the decision to include or exclude articles from the systematic review was made by consensus. The review protocol was not registered in PROSPERO due to organizational constraints.

### 2.3. Data Extraction and Harmonization

Based on PRISMA, a data extraction form was developed [25]. The following information was obtained from each manuscript: authors’ name and year of publication, study design, country, sample characteristics (number of participants, gender, age), the instruments for assessing PF levels, the instruments for assessing active travel or active commuting, main results, and investigation quality. The extraction was achieved by one author (D.H.-N.), and coding was verified by two authors. (S.G.; P.S.-M.)

### 2.4. Study Quality and Risk of Bias

The Quality Assessment Tool for Quantitative Studies checklist was used to assess the articles’ quality [26]. This checklist includes 19 items, which assessed the following criteria: selection bias, study design, confounders, blinding, data collection methods, withdrawals and dropouts, intervention integrity, and analyses. The 19 items were divided in the 8 sections listed above and for each section a score of strong, moderate, or weak methodological quality was given. From the interpretation of the scores of each section, an overall score was given to each article. The study quality and risk-of-bias procedure was performed by two authors (S.G.; A.M.).

### 2.5. Synthesis of Results

This systematic review examined the association between PF and active travel/commuting in young and adult populations. A synthesis of the results and characteristics (such as, design, participant characteristics and sample size, measures, main results, and investigation quality) of each included article are presented. A narrative review of the included studies was performed. 

## 3. Results

### 3.1. Search Results

**Figure 1:**
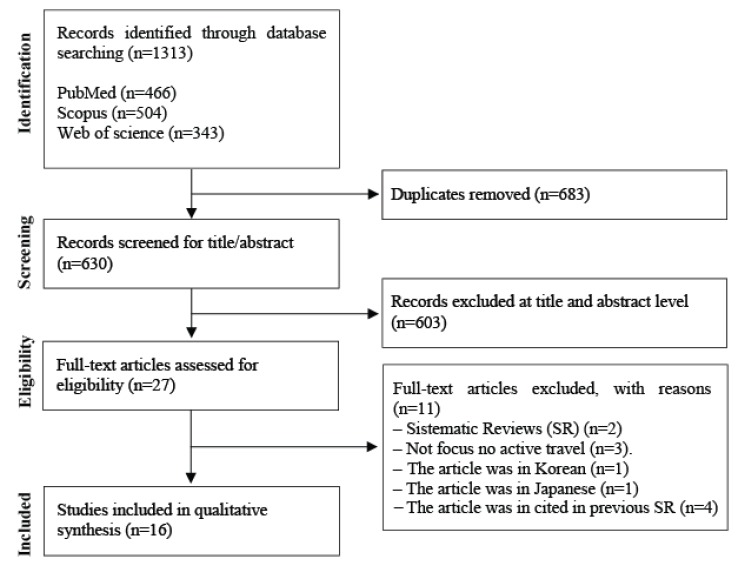
Flow diagram of investigation selection.

A total of 1313 articles were identified during the search. Of those, 683 were identified as duplicates, resulting in 603 articles for the title and abstract screening. In this phase, 27 articles were extracted for full text read, from which 11 were excluded for not meeting the inclusion criteria, namely: three were not focused in active travel/commuting, two were systematic reviews, four were cited in previous systematic reviews, and two were written in Korean or Japanese. Thus, 16 articles were identified as relevant. The flow chart of study selection is presented in Figure 1.

### 3.2. Investigation Characteristics

Table 1 and Table 2 present the characteristics of the studies for children/adolescents and adults, respectively. Seventeen articles were included for final qualitative analysis, with 11 studies targeting the young population, i.e., children (up to 13 years old) and adolescents (up to 18 years old), and five studies that focused on the adult population. Every study was either focused on children/adolescents or on adults. None of the included studies were focused on children/adolescents and adults at the same time. All studies were mainly completed in Europe, for both populations.

#### 3.2.1. Children/Adolescent

From the 11 studies focused on children/adolescents, four were performed in Spain, two in England, two in Norway, one in Sweden, one in Brazil, and one in Colombia. Furthermore, eight were cross-sectional [3,12,27,28,29,30,31,32], one prospective cohort [33], one quasi-experimental [34], and one experimental [35]. The CRF was the PF attribute assessed the most (nine studies), while MF was assessed in three studies [3,27], and agility in two studies [3,31]. Only one study assessed the speed [31]. Active commuting was reported by the participants in most studies (ten studies), except for one study in which the parents reported how their child usually went to school [29]. Distance from house to school, used in one study, was calculated by Google Maps. Seven studies showed a positive association between PF levels and active commuting, mainly in participants who cycled. Two studies observed a positive effect of active commuting on PF attributes assessed in girls but not in boys, and the other five studies found no relationship between active commuting with body composition, CRF, upper strength, and lower strength. Only two studies reported results related to body composition variables. From 11 investigations subjected to methodological quality analysis, one strong, nine moderate, and one weak investigation were identified (Table 1).

In six studies activity, commuting was positively associated with PF [12,27,28,30,33,35]. Active commuting by cycling improves CRF [12,30,35], body composition [12], and muscular strength [27]. One experimental investigation [35] concluded that active commuting improves the CRF, while another quasi-experimental investigation [34] did not find associations between active commuting and PF. The only prospective investigation screened showed that active commuting by cycling in children over a span of six years, increased the PF in 14% [33]. On the other hand, in four studies an association between active commuting and PF was not observed [3,29,32,34]. In one study, mixed results were observed. Girls who actively commuted to school showed better levels of upper limb strength and velocity. However, non-significant associations were observed in boys [31].

#### 3.2.2. Adults 

Results of the studies in adult are presented in Table 2. For adults, five studies were identified: two in Denmark, one in Belgium, one in Finland, and one in Switzerland. From these five studies, four were experimental and two cross-sectional. The CRF was assessed in all studies, while MF was assessed in only one study. Variables of active commuting were assessed mainly by self-report, GPS (global position system), and Google Maps. From the 5 studies with adults, two were classified as having strong methodological quality, one moderate, and two weak methodological qualities. In general, a positive effect of active commuting on PF attributes was observed. Cycling to work has the potential to increase physical performance in an untrained people [36], and bicycle commuting improve CRF and reduced body fat [13,24,37]. Four weeks of active commuting can lead to improvements in CFR [38]. 

## 4. Discussion

The aim of this systematic review was to examine the association between PF and active travel/commuting in young and adult populations. Studies published until December 2019 were identified according to the inclusion criteria. A total of 16 studies were systematically reviewed. Some studies, in young and adult samples, demonstrated that active commuting is related to PF levels. However, in young populations, four studies did not find positive effects of active commuting on PF levels. Overall, the results between active commuting and PF levels in adults seem to be more consistent than those at young ages.

### 4.1. Children/Adolescents

Firstly, CRF is an attribute with a higher genetic component and increases only 8%–9% with three weekly bouts of 20 min of PA, at 80%–90% of maximum heart rate, for 10–12 weeks [39]. Secondly, some tests are flawed, with respect to the estimation of peak VO_2_ in mL/kg/min at young ages [40]. Other factors that can explain the non-association between active commuting and CRF in young ages are as follows: The age of participants, mode of active commuting (e.g., walking or cycling), lower active commuting distance, high-deprivation neighborhoods, frequency of commuting, and the overall amount of time spent being an active commuter [12,28,30,33]. When examining the association between active commuting and MF, the authors observed that cyclists had higher handgrip strength and walkers had higher vertical jump peak power when compared with non-active commuters [27]. Furthermore, positive association was observed between active commuting and upper limb strength in girls but not in boys [31]. This result is crucial to retain, in order to understand the various impacts of active commuting on girls and boys. Different results in PF between girls and boys can be explained because the girls usually have lower levels of PA and MF than boys. The specific type of physical exercise (walking or cycling) promotes specific physiologic adaptations, which act in several intensities on several attributes of PF [41,42].

The positive association between active commuting and CRF in prospective study [35] is in accordance with previous investigations, which concluded that promoting active commuting to school among children and adolescents may be a useful strategy to improve CRF and other health outcomes [11,43,44]. Our discoveries highlighted the variability of the investigations’ results, which can be explained by different methodologies applied and/or non-control of other confounders. The social environment and the neighborhood characteristics are crucial factors to be considered when promoting active commuting in youth [30]. Overall, active commuting is associated with healthier levels of PF among youth. The findings from this systematic review concur with those from previous systematic reviews [18,19].

**Table 1:** Characteristics of the studies in children and adolescents.

Author, Year	Study Design	Country	Sample	Physical Fitness Attribute (Measure)	Active Commuting Measure	Observation	Main Results	Study Quality
Børrestad et al., 2012 [35]	Experimental	Norway	Total n = 204 IG, 26 (10.8 ± 0.7 years), Boys (53.9%) CG, 27 (10.9 ± 0.7 years), boys (51.9%)	CRF: Peak oxygen consumption (VO_2peak_, mL O_2_/min/kg), HR_peak_ (h/min), BMI (kg/m^2^)	Participants reported how many days a week they traveled to/from school in the last 3 months by walking, cycling, car, or public transport. Distance to school (km).	Active commuting; Cycle ergometer test	Active commuting by cycling in both groups (IG and CG) improves the CRF in children.	Moderate
Chillón et al., 2012 [33]	Prospective cohort	Sweden	Total n = 262 120 boys, 142 girls Swedish children who were involved in the European Youth Heart Study (EYHS)	CRF: (VO_2max_) expressed in absolute terms (L/min); BMI (kg/m^2^); WC (cm); Skinfolds (mm)	Participants reported how they go to school. Passive: car, bus, train or Active: bicycle or walk (%)	Active commuting; Cycle ergometer test; calipers	Bicycling to school in childhood was related to improvements in fitness 6 years later. Children who became bicyclists in adolescence improved their fitness levels. No changes were observed for fatness.	Moderate
Østergaard et al., 2013 [12]	Cross-sectional	Norway	Total n = 1694, aged 9–15 years, 577 Boys, 482 Girls Norwegian who were participated in the Physical Activity among Norwegian Children Study	CRF: (VO_2max_, mL/kg/min); Functional strength (cm), Muscular endurance (n) (s); BMI (kg/m^2^); Skinfolds (mm)	Participants reported how they go to school: passively (car/ motorcycle or bus/train) or actively (bicycle or walk).	Active commuting Time of travel (minutes); Cycle ergometer test; Standing jump, Sit-ups, Biering–Sørensen test, Harpenden calipers	Active commuting, especially cycling, is positively associated with body composition, CRF, and MF when compared to passive commuting.	Strong
Ropero et al., 2014 [27]	Cross-sectional	England	Total n = 6829, aged 10–16 years; (53% males, age 12.9 ± 1.2 years) English adolescent who participated in the East of England Healthy Hearts Study	Muscular fitness: upper strength (kg), lower strength (cm) and (W·kg^−1^); BMI (kg/m^2^)	Participants reported how they go to school: passively (car or public transport) or actively (bicycle or walk. Distance to school (km).	Active commuting: Distance from home to school calculated by Google Maps. MF: Handgrip test, Vertical jump	When compared with passive travelers, cyclists had higher handgrip strength and walkers had higher vertical jump peak power.	Moderate
Villa-González et al., 2015 [28]	Cross-sectional	Spain	Total n = 494, aged 8–11 (9.2 ± 0.6) years, 577 Boys (9.3 ± 0.6 years), 229 (9.2 ± 0.6 years) Girls.	CRF (VO_2max_ mL·min^–1^·kg^–1^, stage); MF (cm, kg), Agility (s).	Participants reported how they go to school: passively (car or public transport) or actively (bicycle or walk).	Active commuting Weekly frequency: (0–2 active travels vs. 3–7 active travels vs. 8–10 active travels); PACER test, Push-up test, Handgrip test, Standing long jump, Leg extension test.	No associations were found between active commuting with CRF and upper body MF. Positive associations between active commuting with agility and lower body MF in girls and boys.	Weak
Noonan et al., 2017 [30]	Cross-sectional	England	Total n = 194, aged 8–11 (9.2 ± 0.6) years, 87 Boys (9.97 ± 0.30 years), 107 Girls (9.95 ± 0.30 years).	CRF (laps); MF: upper strength (kg), lower strength (cm) and (W·kg^−1^); BMI (kg/m^−2^)	Participants reported by how they go to the school: passively (scooter, bus, car, train, taxi, other) or actively (bicycle or walk).	Active commuting; distance (km) calculated by Google Maps; PACER test, Push-up test, Handgrip test, Standing long jump, Leg extension test.	Active commuters, who live further away from school had better cardiorespiratory fitness.	Moderate
Pires et al., 2017 [31]	Cross-sectional	Brazil	Total n = 751, aged 7–17; 312 Boys and 349 Girls.	MF: upper strength (m), lower strength (m); Speed (s); Agility (s) BMI (kg/m^2^)	Participants reported how they go to school. Passive: car, bus, train or active- bicycle, walk (%)	Active commuting (%); Medicinal ball throw; Standing long jump; Square test.	Girls who actively commute to school showed better levels of upper limb strength and velocity. No significant difference was observed for the physical fitness between transport groups in boys.	Moderate
Villa-González et al., 2017 [34]	Quasi-experimental	Spain	Total n = 251, aged 8–11 (9.2 ± 0.6) years, IG: 73 boys and 68 girls; CG: 54 boys and 56 girls.	CRF (VO_2max_ mL/kg/·min, stage); MF (cm, kg), Agility (s).	Participants reported how they go to school. Passively (car, bus, train) or actively (bicycle, walk).	Weekly frequency (0–2 active travels vs. 3–7 active travels vs. 8–10 active travels); PACER test, Push-up test, Handgrip test, Standing long jump, Leg extension test.	No associations between active commuters and health-related fitness.	Moderate
Ramirez-Veléz et al., 2017 [3]	Cross-sectional	Colombia	Total n = 2877, aged 7–17, 312 boys, 349 girls.	CRF Peak oxygen consumption (VO_2peak_ mL/O_2_/min/kg); MF: upper strength (kg), lower strength (cm); Flexibility (cm); Agility (s); BMI (kg/m^2^), WC (cm).	Participants reported how they go to school: by car, public transportation or actively (walking, cycling).	Active commuting (days per week); PACER test; Handgrip test; Standing long jump test; 4 × 10 m shuttle run.	Regular cycling to school may be associated with better physical fitness, especially in girls.	Moderate
Muntaner-Mas et al., 2018 [32]	Cross-sectional	Spain	Total n = 2518, aged 10–16 years (13.0 ± 2.1).	CRF (VO_2 peak_, mL kg min^−1^); BMI (kg/m^2^).	Participants reported how they go to school. Passively (car, bus, train) or actively (by bicycle, walk, by riding skate).	Active commuting (%); PACER test.	No relationship between active commuting to school and CRF in children and adolescents.	Moderate
Ruiz-Hermosa et al., 2018 [29]	Cross-sectional	Spain	Total n = 2518, aged 4–7 years (13.0 ± 2.1).	CRF (VO_2_ peak, mL kg min^−1^); MF: lower strength (cm); BMI (kg/m^2^), WC (cm), Skinfolds (mm).	Children’s parents reported how they go to school. Passive (car, bus, train) or Active (bicycle, walk)	Active commuting (time); Course-Navette or PACER test; Standing long jump test; Holtain Ltd. Caliper	No relationship between walking to school with adiposity indicators, physical fitness.	Moderate

BMI, body mass index; CG, control group; CRF, cardiorespiratory fitness; IG, intervention group; MF, muscular fitness; MOD, moderate activity; PACER, Progressive Aerobic Cardiovascular Endurance Run; VIG, vigorous activity; WC, waist circumference.

**Table 2:** Characteristics of the studies in adult.

Author, Year	Study Design	Country	Sample	Physical Fitness Attribute (Measure)	Active Commuting Measure	Observation	Main Results	Study Quality
De Geus et al., 2009 [36]	Experimental	Belgium	Total n = 80 IG, 30 males (43 ± 6 years), 35 females (43 ± 3); CG, 7 males (50 ± 8 years), 8 females (48 ± 6 years)	CRF (Maximal external power [P_máx_ (/kg)]; Peak oxygen uptake [VO_2peak_ (/kg)], Absolute maximal external power (P_máx_), Relative peak oxygen uptake (VO_2peak_/kg), Heart ratio max (beats/min), respiratory exchange ratio (VCO_2_/VO_2_)	Participants reported a weekly diary. Distance and the time spend on each trip by car/motorcycle; bus/train; bicycle; walk to work.	Measured the distance and the time spend on each trip; cycle ergometer test.	The maximal external power and peak oxygen uptake increased significantly in IG (Male and Female). Cycling to work has the potential to increase physical performance in an untrained study population.	Moderate
Moller et al., 2011 [37]	Experimental	Denmark	Total n = 48 IG 13 males (43 ± 8.9 years), 6 females (44.4 ± 8); CG, 16 males (46.1 ± 9.9 years), 7 females (46 ± 9.1 years	CRF (VO_2max_ ml/kg/min); Heart ratio max (beats/min); Respiratory exchange ratio (VCO_2_/VO_2_); BMI (kg/m^2^); Skinfolds (mm)	Participants used their bicycle and registered the cycling distance	Active commuting was calculated by (Mavic M-Tech 7) Cycle ergometer test; Harpenden calipers	CRF was significantly improved and body fat reduced in 8 weeks of commuter cycling.	Strong
Vaara, et al., 2014 [13]	Cross-sectional	Finland	Total n = 781, aged 18–90 years (47.1 ± 8.7 years); Male (81.9%)	CRF: VO_2max_, mL/kg/min. MF: reps/min, kg and N). WC (cm), body fat: bioelectrical impedance.	Participants reported a weekly diary. The time spend per day by bicycle or walk to work	Active commuting was classified by total time. CRF was assessed by cycle ergometer, and VO_2max_ estimated from HR and maximal power.	The high active commuting group showed better results in CRF, some MF tests and WC with other active commuting groups.	Weak
Hochsmann et al., 2018 [38]	Experimental	Switzerland	Total n = 32 adults, aged 18–50 years. 28 males and 2 females	CRF: (VO_2 peak_, mL kg min^−1^); BMI (kg/m^2^).	E-bike group and bike group reported a typical route to work.	Active commuting (km and elevation calculated by Google Maps, Google Inc, Mountain View, California). CRF was assessed by cycle ergometer.	A period of 4 weeks of active commuting can lead to improvements in VO_2peak_ in both groups. Moreover, no significant difference in VO2_peak_ and maximal ergometric workload gain.	Weak
Blond et al., 2019 [24]	Experimental	Denmark	Total n = 130 adults, aged 20–45 years. CG 18 (male 9, female 9); IG bike 35 (male 16, female 19); IG/MOD 39 (male 19, female 20); IG/VIG 38 (male 20, female 18)	CRF: (VO_2 peak_, mL kg min^−1^); BMI (kg/m^2^).	The daily distance was calculated for participants in bike based on their energy expenditure while cycling from/to work/school.	The active commuting distance was monitored using Polar RC3 GPS (Polar, Finland). CRF was determined using an electronically braked cycle and open circuit indirect respiratory calorimetry.	CRF increased in all exercise active commuting groups compared with non-active commuting.	Strong

BMI, body mass index; CG, control group; CRF, cardiorespiratory fitness; IG, intervention group; MF, muscular fitness; MOD, moderate activity; VIG, vigorous activity; WC, waist circumference.

### 4.2. Adults

From the five studies screened, a positive association between active commuting and several PF attributes was observed. A cross-sectional investigation showed a positive association between active commuting and metabolic health, along with the beneficial impact on the promotion of the PA levels in adults. However, no association between active commuting and CRF and MF was found [13].

Four intervention investigations used cycling to analyze the potential positive effects of active commuting on PF levels. After one year of experimental investigation, the authors concluded that cycling to work had a positive effect on CRF levels on the intervention group [36]. The experimental investigation, performed over six months, showed active bike commuters presented better CRF than the non-active commuting group, but not when compared with the group that performed vigorous PA in leisure time [24]. Both active groups had similar improvements [24]. However, it seems that for the same period of intervention, the intensity of PA plays an essential role in the improvement of CRF [45]. The majority of studies performed in adults indicated that active commuters had greater cardiovascular fitness, especially those who cycled to and from work. Cycling to and from work seems to be an essential tool to reduce the time required to expend a given quantity of energy. Additionally, high-intensity physical exercises seem to be a fundamental exercise component to increase CRF, and they serve as a protective factor against several metabolic diseases [24,45,46]. Active commuting improved the cardiometabolic health and CRF in both groups, but with slow effects in the active commuting group when compared with the leisure-time vigorous-intensity group [24]. Previous investigations have shown the impact of lifestyle exercise on adiposity, while the changes in CRF seem to be more dependent on the exercise intensity [45,47].

Several limitations have been identified in these investigations, which can explain the results. However, it was possible to identify potential mediators or confounders, which can influence the relationship between active commuting and the results of several attributes of PF [13,23]. Although most studies examined the effects of active commuting and non-active commuting, an experimental investigation analyzed the effect of E-Bike versus bike commuting on CRF in overweight adults, which concluded that both bike systems increased CRF levels, even if the bikes were electrically assisted [38].

Ambiguous or inconsistent results observed mainly among children and adolescents in this systematic review can be the result of the several methodologies used to access PF attributes, the type of active commuting, the type of population, the geographic area, and the environmental context. Additionally, the measures of active commuting extensively varied (e.g., frequency, duration, and distance) among the investigations screened in this systematic review. Due to all these factors, rigorous comparison among investigations is highly limited.

This systematic review is not without some limitations. Firstly, active travel/commuting was self-reported in all the included studies. This may be subject to bias, especially at young ages. Secondly, most investigations do not control the intensity of active commuting. Thirdly, studies were mainly focused on a specific world area in high- or mid-income countries. Finally, the terms selected to identify investigations that examined active commuting and PF could have excluded several articles (e.g., ones in which the predefined terms were found neither in the title nor the abstract).

Overall, the results seem to indicate that active travel/commuting and PF are positively associated in young and adult population. Walking and cycling are common modes used by young active commuters. Young cyclists had higher CRF level, while walkers had better MF levels. In adults, cycling is the principal mode of active commuting and is associated with greater PF levels, especially CRF. Active commuting by cycling increases the intensity level of physical activity and seems to be an excellent strategy to improve the PF levels. These findings highlight that active commuting promotes health status. However, for all entities who promote active commuting, it is essential considering factors such as age, sex, and environment at the moment to select the adequate active commuting mode.

## 5. Conclusions

Findings from this review suggest that among younger ages, active travel/commuting is inconsistently related to PF and that several factors should be considered to compare the effectiveness of active commuting in improving PF outcomes in children and adolescents. Findings of studies in adults demonstrated the positive effect of active commuting on CRF and body composition. Overall, most studies have shown a positive relationship between active commuting and several attributes of PF. Additionally, active commuting, by cycling, seems to have a more positive impact on several PF attributes. However, there is a need to identify potential mediators or confounders in this relationship, in order to avoid misconceptions. More investigations on this topic are essential to understand how and whether decision makers and public health authorities can use active travel/commuting as a strategy to improve PF in all ages. In order to achieve that, it is important that future investigation pursue a more detailed approach when examining active travel/commuting, especially taking into account its context and specificity.
